# The preference for water nipples vs. water bowls in dairy goats

**DOI:** 10.1186/1751-0147-53-50

**Published:** 2011-09-22

**Authors:** Knut E Bøe, Rebecca Ehrlenbruch, Inger L Andersen

**Affiliations:** 1 Norwegian University of Life Sciences, Department of Animal- and Aquacultural Sciences, P.O. Box 5003, 1432 Ås, Norway

**Keywords:** goat, water dispenser

## Abstract

**Background:**

Previous studies have reported that the design of the water dispensers can influence the water intake in farm animals. Horses and dairy cows seem to prefer to drink from an open surface whereas sheep and pigs apparently prefer water nipples, probably because of the worse water quality in water bowls. The aim of the present study was to examine the preference of dairy goats for water nipples or water bowls.

**Methods:**

In each of the two experiments (exp. 1, dry goats, exp. 2 lactating goats), 42 dairy goats were allotted into 6 groups of 7 goats. In period 1, the goats had access to a water nipple. In period 2, they had access to a water bowl and in period 3 (preference test) they had access to both a water nipple and a water bowl. Water usage and wastage was recorded and water intake (water usage - water wastage) was calculated for each group for the two last days of each period. In experiment 2, water samples from each dispenser were analyzed for heterotrophy germs at 22°C, *Escherichia coli *and turbidity.

**Results:**

Water usage was higher from water nipples than from water bowls both in experiment 1 (dry goats) and experiment 2 (lactating goats). There was however, no difference in water intake from water nipples and water bowls. In the preference test (period 3), the water intake tended to be higher from the water nipple than from the water bowl both for the dry goats (exp. 1) and lactating goats (exp. 2). Especially for the dry goats, the differences between groups were large. Turbidity and heterotrophy germs were much higher in the samples from the water bowls than from the water nipples.

Water wastage from the water bowls was negligible compared to the water nipples. From the water nipples the water wastage was 30% and 23% of water usage for the dry and lactating goats respectively.

**Conclusions:**

We conclude that type of water dispenser (nipple or bowl) was probably of minor importance for water intake in goats, but water bowls had a lower water quality.

## Background

The literature on water intake in goats is scare, and the studies mainly concern goats living in desert conditions under heat stress and/or water restrictions [e.g. 1]. In temperate climates, the water intake for goats is reported to be 139 g/kg W^0.75 ^at mid-pregnancy, and lactating goats need 1.28 kg of water to produce one kg milk [2]. Ehrlenbruch et al. (2010) measured the water intake in lactating goats to be 6.2 and 4.4 liters/day when fed hay and silage, respectively [3].

Previous studies have reported that the design of the water dispensers can influence the water intake, and many farm animals species seem to prefer to drink from a water source with a large and open surface (horses: [4]; cows: [5,6]). However, both in pigs [7,8] and in sheep [9] the water intake was higher from nipple drinkers than from water bowls. The water quality seems to be important for the lower intake from water bowls, and both Bøe (1984) and Bøe and Kjelvik (2011) reported a clear reduction in water quality in water bowls [9,10]. Brooks and Carpenter (1989) found that in weaned piglets, the water intake from bowls declined when the water became fouled with feed or feces [11]. To our knowledge, there is at present no data on preference for type of water dispenser in goats, but a survey of Norwegian goat herds showed that use of water bowls had a negative effect on both somatic cell count and bacterial count in the goat milk [12].

Water wastage seems to be much higher on nipple drinkers than on different types of water bowls both in pigs [7,8,13] and in sheep [9]. The amount of water wastage from nipples can be somewhat reduced by adjusting the nipple heights to the shoulder height of the pigs and by reducing the flow rate [13,14]. High amounts of water wastage will increase the water usage, impair the quality of the lying surface in pens with bedding and increase the necessary volume of manure storage.

The aim of the present study was to examine the preference in dairy goats for water nipples or water bowls. Based on previous studies in sheep and pigs, we predicted that goats would prefer to drink from water nipples and that the water quality will be worse in water bowls.

## Methods

### Experimental design

In each of the two experiments, six groups of seven dairy goats were allotted to experimental pens with one water nipple and one water bowl for 12 days. During period 1 (4 days) the goats had only access to the water nipple. In period 2 (4 days) they had only access to the water bowl, and during the actual treatment in period 3 (4 days), the goats had access to both the water nipple and the water bowl.

### Water equipment and experimental pens

**Figure 1:**
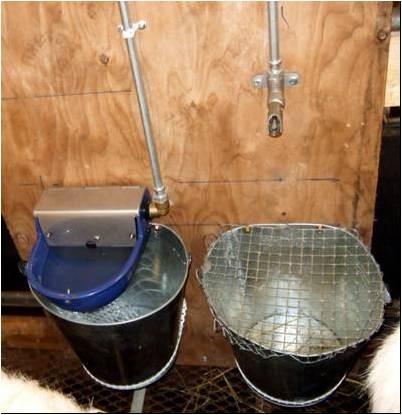
**Water nipple and water bowl with a container under each water dispenser to collect water wastage**.

The experiments were conducted in an insulated, mechanically ventilated building where the average air temperature was kept at 10-14°C. Each group of goats was kept in pens with expanded metal flooring and with a total area on 5.0 m^2 ^giving 0.83 m^2^/goat. One bowl (automatic float valve CF7, art. no.: 972 824 90, DeLaval^® ^) and one nipple (Nipple Drinker mod.293, Suevia Haiges^® ^) were installed in each pen 0.58 m and 0.75 m above floor level, respectively (Figure 1), and had a flow rate of approximately 3.0 l/min.

### Animals and feeding

In each of the two experiments, 42 goats of the Norwegian dairy breed were used, giving a total of 84 animals. In experiment 1 (performed in January), the goats were dry and about 60 days pregnant, and were on average 3.5 years old (range 2-9 years) with a body weight of 61.1 ± 1.1 kg (mean ± SE). The goats in experiment 2 (performed in late November) were in late lactation with an average daily milk yield of 1.6 ± 0.1 kg (mean ± SE). These goats were on average 4.2 years old (range 2-10 years) and had a body weight of 58.3 ± 0.9 kg (mean ± SE). From May to September the goats were on pasture and here the water were supplied in standard water bowls, while during the indoor feeding period (October to May) the water supply was nipple drinkers.

The goats were offered hay *ad libitum *and 0.4 kg and 1.2 kg per day of a standard concentrated diet in experiment 1 and 2 respectively. In experiment 2 the goats were milked twice a day (0700-0800 and 1500-1600 hours).

### Recording of water usage, water wastage and water intake

Water meters (Altaïr N°C05 A4) were connected to the water supply pipeline for the water nipple and water bowl in each pen (accuracy ± 0.1 litre). A container with the top made of metal grids was located below each water dispenser to collect water wastage (Figure 1). Every morning at feeding (0730 hours), the water usage was recorded by reading the water meters, and the water wastage in the containers were weighed on an electronic balance. The water intake of the group was then calculated as water usage minus water wastage for each day. The mean for the two last days of each experimental period was calculated and used as statistical unit in the statistical analysis.

In order to characterize a clear preference for type of water dispenser a cut-off point on ≥ 70% of the total water intake (on both dispensers, period 3) was set.

### Behavioural observations

In experiment 2, each goat was individually marked on its back. A video camera was suspended above each pen and directly connected to a computer using the MSH video system^® ^http://www.guard.lv. All pens were videotaped the last 24 h of period 3. Start time (when the goat had its lips round the nipple or the mouth into the water surface in the bowl) and stop time (when the goat moved the lips or mouth away from the dispenser) for every drinking bout for each goat was scored continuously for 24 h with one second accuracy. From these observations the individual drinking frequency and drinking time (sum of duration of each drinking bout) on each dispenser was calculated. Also here, to characterize the individual preference for type of water dispenser we chose to set criteria to ≥ 70% of total drinking time on one of the dispensers.

### Water quality

In experiment 2, double water samples from each dispenser were taken on the last day in period 3, and the water samples were purred into sterilized and sealed plastic bottles. A control sample was taken from a sterilized water tap in the milking room. The samples were analyzed for heterotrophy germs at 22°C (CFU/ml, method: NS-EN ISO 6222), *Escherichia coli *(CFU/100 ml, method: NS-EN ISO 9308-1) and turbidity (FNU, method: ISO 7027).

### Ethical note

A university representative of the National Research Authority http://www.fdu.no9 approved this experiment, and no ethical concerns were indicated.

### Statistical analysis

The effect of type of water dispenser (water nipple and water bowl) on water usage, water intake and water wastage was analysed using Wilcoxon signed rank test. The water intake from the two dispensers (period 3) and individual drinking time and duration of drinking bouts (experiment 2, period 3) were also analysed using a paired comparison t-test.

Data on individual drinking time and duration of drinking bouts (experiment 2, period 3) were analyzed using a mixed model analysis of variance with type of water dispenser as fixed effect, and group was specified as a random effect in the model.

## Results

### Water intake and water wastage when access to either nipple or bowl (period 1 and 2)

Water usage was higher from water nipples than from water bowls (Table 1) both in experiment 1 (dry goats) and experiment 2 (lactating goats). There was however, no difference in water intake from water nipples and water bowls.

**Table 1:** Water usage (l/goat and day), water intake (l/goat and day), and water wastage (% of usage) when access to either nipple (period 1) or bowl (period 2) (mean ± SE) in dry (exp. 1) and lactating (exp. 2) goats

	Water nipple	Water bowl		
Experiment 1			t	P
Water usage	3.51 ± 0.20	2.42 ± 0.19	4.09	< 0.01
Water intake	2.45 ± 0.08	2.41 ± 0.19	0.18	ns
Water wastage (% of water usage)	30.2 ± 2.5	0.6 ± 0.2	12.08	< 0.0001

Experiment 2			t	P
Water usage	5.21 ± 0.16	4.26 ± 0.26	4.81	< 0.05
Water intake	4.00 ± 0.18	4.26 ± 0.26	-1.87	ns
Water wastage (% of usage)	23.2 ± 1.9	0.1 ± 0.0	12.25	< 0.0001

Water wastage from the water bowls was negligible compared to the water nipples (Table 1). From the water nipples the water wastage was 30% and 23% of water usage for the dry and lactating goats respectively.

### Preference for water nipple or water bowl (period 3)

In experiment 2 (see Table 2) the water intake tended to be higher from the water nipples than from the water bowls (t_5 _= 2.50, P = 0.07). Three of the groups in experiment 1 showed a clear preference for the water nipple (≥ 70% of water intake) and one group for the water bowl. In experiment 2, only one group showed a clear preference for the water nipple (≥ 70% of water intake).

**Table 2:** Water intake from the water nipple and the water bowl and proportion of water intake from water nipple when access to both (preference test, period 3)

	Exp. 1			Exp. 2		
	
Group	Water nipple (l/goat and day)	Water bowl (l/goat and day)	Water nipple (% of intake)	Water nipple (l/goat and day)	Water bowl (l/goat and day)	Water nipple (% of intake)
1	2.15	0.14	94.1	2.23	1.60	58.2
2	1.91	0.39	83.2	1.75	1.85	48.6
3	1.15	1.40	45.1	2.72	2.50	52.1
4	0.74	1.59	31.8	2.13	1.33	61.5
5	2.03	0.50	80.4	2.70	1.66	61.9
6	0.53	2.06	20.5	2.54	1.24	67.2

Mean	1.42	1.01	59.2	2.34	1.69	58.3

### Individual preferences

Mean total drinking time was significantly longer on the water nipple than on the water bowl (mean ± SE: 149.0 ± 17.8 vs 84.6 ± 15.3 sec, F_1,77 _= 7.56, P < 0.01) and the duration of each drinking bout was shorter when drinking from the water nipple than from the water bowl (mean ± SE: 5.5 ± 0.6 vs 14.7 ± 1.9 sec., F_1,77 _= 22.37, P < 0.001). Of the 42 goats, 23 preferred nipples (≥ 70% of total drinking time), 10 goats preferred the bowls (≥ 70% of total drinking time) and 9 goats did not show a clear preference. In every group there were some goats that preferred the water nipple and some that preferred the water bowl. Four of the 42 goats were never observed drinking from the water nipple and seven goats were never observed drinking from the water bowl.

### Water quality

Turbidity and heterotrophy germs were much higher in the samples from the bowls than from the nipples (Table 3). There was a large variation in turbidity among the samples from groups drinking from bowls (range 1.5-16 FNU), and samples from both group 4 and 6 had turbidity > 12 FNU. *E. coli *was not detected in any of the water samples.

**Table 3:** Water quality in water nipples and water bowls in experiment 2 (mean ± SE)

	Water nipples	Water bowls	Control
Turbidity (FNU)	0.2 ± 0.0	6.4 ± 1.6	0.1 ± 0.0
Heterotrophy germs at 22°C (CFU/ml)	103.0 ± 18.9	> 3 000 *	1.3 ± 0.6
*Escherichia coli *(CFU/100 ml)	0.0 ± 0.0	0.0 ± 0.0	0.0 ± 0.0

* All recordings > 3 000.

## Discussion

As predicted, the goats showed an overall preference for drinking from the water nipples, which is in accordance with previous studies on pigs [7,8] and sheep [9]. The preference for water nipples was however not uniform and total, in that two groups actually showed a preference for water bowls and even in the remaining groups apparently preferring water nipples, a considerable proportion of the water intake was from the water bowl. Also when exploring individual preferences, data revealed that some goats preferred water nipples and some preferred water bowls, also within the same groups. This is in accordance with many previous preference tests that show large differences between individuals [e.g 15]. Ideally, the water nipple and water bowl should have been introduced in randomised order in period 1 and 2, and hence carry-over effects cannot be eliminated. However, since the goats had been exposed to water bowls in the whole grazing season and water nipples in the current indoor feeding period, and the water intake was equal on water nipples and water bowls in period 1 and 2, it seems reasonable to assume that order of introduction of water dispensers did not have a major influence the results. Still, the results should be interpreted with caution.

The water nipple and the water bowl were positioned quite near to each other, which may have triggered social competition, and thus forced individuals with low social rank to drink from another type of water dispenser than they originally preferred. Still, this is less likely with only seven animals per group, as previous experiments with goats showed that only when increasing the number of animals per water nipple to more than 15, the queuing and displacements increased [16].

As predicted, the water quality from the water bowls was clearly inferior to water nipples, which is in accordance with previous findings in pigs [10,11]. Turbidity was higher in the water bowls than what is accepted in human drinking water (< 4 FNU) and also heterotrophy germs at 22°C was higher in the water bowls than recommended (< 100 CFU/ml). This poor water quality is probably the main reason for the goats' preference for water nipples. It is however interesting to notice that the preference for water nipples was not more pronounced in the two groups that experienced the worst water quality. On the other hand, drinking water quality must be of importance in goats since use of water bowls has been documented to increase the somatic cell count and bacterial count in the goat milk [12]. What the goats prefer does thus not necessarily reflect what is the optimal choice in terms of health.

As predicted, the water wastage was quite high from water nipples (23-27% of water usage) and almost negligible from water bowls. This is in accordance with findings both in weaned piglets and growing-finishing pigs [7,8,13] and sheep [4]. It is possible that the amount of water wastage could have been reduced some by altering the mounting heights, a measure that apparently has been partly successful in pigs [13,14]. The amount of spillage from the nipple drinkers represents a considerable amount that has to be stored in the manure storage and thus increase labour input and costs.

## Conclusion

We conclude that type of water dispenser (nipple or bowl) was probably of minor importance for water intake in goats, but water bowls had a lower water quality.

## Competing interests

The authors declare that they have no competing interests.

## Authors' contributions

RE carried out the experiments and participated in preparing the manuscript. KEB planned the experimental design, performed the statistical analysis and participated in preparing the manuscript. All authors contributed to the preparation of the manuscript and also read and approved the final manuscript.
